# Dunglison’s Medical Dictionary

**Published:** 1846-08

**Authors:** 


### DUNGLISON'S MEDICAL DICTIONARY.

This Dictionary has reached the sixth edition, and prefers claims upon the attention and regard of students which cannot easily be resisted. If it is not "*indispensable*" to the profitable prosecution of their studies, it will be found so useful, so satisfactory and desirable, that not many will agree to do without it. In proof of the care bestowed upon this edition of his dictionary, the author remarks that it "contains nearly two thousand five hundred subjects and terms not contained in the last edition." We speak of the merits of this lexicon from long acquaintance with it; and of the many works to which we acknowledge ourselves under a debt of gratitude, there are few of which we should speak in terms of more hearty commendation than the dictionary of Professor Dunglison.

